# ﻿New record of *Gracilariaphuquocensis* (Gracilariaceae, Rhodophyta) in the Philippines

**DOI:** 10.3897/phytokeys.241.123302

**Published:** 2024-04-29

**Authors:** Richard V. Dumilag, Lawrence M. Liao, Aki Kato, Juliet Brodie, Narongrit Muangmai

**Affiliations:** 1 Graduate School, Sorsogon State University, Sorsogon City Campus, Magsaysay St., Salog (Poblacion), Sorsogon City, 4700, Philippines; 2 Graduate School of Integrated Sciences for Life, Hiroshima University, 1-4-4, Kagamiyama, Higashi-Hiroshima, 739-8528, Japan; 3 Fisheries Laboratory, Blue Innovation Division, Seto Inland Sea Carbon-neutral Research Center, Hiroshima University, Minato-Machi, Takehara, Hiroshima, 725-0024, Japan; 4 Natural History Museum, Research, Cromwell Road, London, SW7 5BD, UK; 5 Department of Fishery Biology, Faculty of Fisheries, Kasetsart University, Bangkok 10900, Thailand; 6 Biodiversity Center, Kasetsart University (BDCKU), Bangkok 10900, Thailand

**Keywords:** Agarophyte, distributional records, *Gracilariababae*, *rbc*L, taxonomy

## Abstract

While reliance on morphology has been at the expense of clearly distinguishing gracilarioid species, molecular data have proven to be more reliable in discriminating between taxa. *Gracilariaphuquocensis* was originally described, based on materials collected from Vietnam. Since it was described in 2020, there have been no further reports of this species. Meanwhile, a question has been raised as to whether the identity of a rhodophyte gracilarioid alga collected from the Philippines that has been referred to as an unidentified species of *Gracilaria*, could be *G.phuquocensis*. Based on comparative morpho-anatomical features and a molecular phylogeny based on *rbc*L gene sequences, establishing the identity of the Philippine material has led to the finding of the new record of *G.phuquocensis* outside its type locality. In addition to the discovery of *G.phuquocensis* in the Philippines, the species here is also identified as a newly-reported host for the adelphoparasite resembling *Gracilariababae*.

## ﻿Introduction

Members of *Gracilaria* Greville are a major source of agar, a valuable substance widely used in various industries (Torres et al. 2019; Mantri et al. 2023). The genus has over 270 currently-recognised species worldwide (Guiry and Guiry 2023), a number that continues to increase with new taxonomic work (Freshwater et al. 2022; Saengkaew et al. 2022; Wang et al. 2023). As gracilarioids have relatively low morphological diversity and are speciose, discrimination between taxa is better achieved using combined morphological and genetic data (Lyra et al. 2015; Gurgel et al. 2018; Lyra et al. 2021).

*Gracilariaphuquocensis* N.H. Le, N. Muangmai, G.C. Zuccarello was established based on a specimen collected from Phu Quoc Island in Vietnam (Le et al. 2020). The application of the name became important for the taxonomic clarification of some flattened gracilarioids, which vary in blade dimensions, number of medullary cell layers and tetraporangial features. The use of morphology in combination with molecular data indicates that *G.phuquocensis* is a separate species. However, a question remained regarding the distribution of *G.phuquocensis* beyond its type locality. Using a molecular-assisted alpha taxonomic approach, here, we confirm the presence of *G.phuquocensis* in the Philippines.

## ﻿Materials and methods

### ﻿Specimens collection

With the aim of exploring the diversity of *Gracilaria*, a recent collection of flattened gracilarioids was made in Bulusan (12°43'N, 124°08.3'E), Sorsogon, Philippines. The specimens were processed as dried herbarium voucher specimens and housed at the Natural History Museum of the National Science Museum (THNHM), Thailand and at the Herbarium Sorsogonense (HS), the Philippines. Morphological characters of the thallus in cross section were examined by light microscopy.

### ﻿DNA extraction, PCR amplification and sequencing

For molecular analyses, total DNA was extracted from apical portions of dried algal specimens using a QIAGEN DNeasy Plant Mini Kit (QIAGEN, Hilden, Germany). The ribulose-bisphosphate carboxylase (*rbc*L) gene was selected for PCR amplification as this is a powerful molecular marker for phylogenetic analyses in *Gracilaria* (Gurgel 2002; Gurgel and Fredericq 2004). PCR amplification profile and procedure followed Muangmai et al. (2014). All amplified products were cleaned and sequenced commercially (U2Bio Inc., Seoul, South Korea). Sequences were edited, assembled, and aligned using the Geneious Prime software package (Biomatters, available from http://www.geneious.com/).

### ﻿Phylogenetic analyses

Phylogenetic trees were reconstructed using Maximum-Likelihood (ML) and Bayesian Inference (BI) methods using IQ-TREE (Trifinopoulos et al. 2016) and MrBayes v.3.2 (Ronquist et al. 2012), respectively. ML analyses were carried out under GTR+R model, using IQ-TREE web server (http://iqtree.cibiv.univie.ac.at) under default option with 1000 bootstrap replicates. BI analyses were performed using a GTR + I + R model, with two parallel runs of four Markov chains for a million generations. We sampled one tree every 1000 generations and then removed 125 trees (burn-in) before determining a consensus topology. Both ML and BI trees were edited with the programme FigTree v.1.4.3 (Rambaut et al. 2018).

## ﻿Results

### ﻿ Gracilaria phuquocensis

Taxon classificationPlantaeGracilarialesGracilariaceae

N.H. Le, N. Muangmai & G.C. Zuccarello, 2019

139310FE-A07A-534E-BFEE-BB8BDCCE92C5

Fig. 1

#### Specimens examined

. Philippines • Sorsogon, Brgy Dancalan, in front of Villa Luisa Celeste Resort; 28 February 2023; N. Muangmai leg.; THNHM-P-2023-0089 (A563), GenBank: OR427952; THNHM-P-2023-0090 (A585), GenBank: OR427953; THNHM-P-2023-0091 (A586), GenBank: OR427954; THNHM-P-2023-0092; THNHM-P-2023-0093; • Dancalan Beach, 28 February 2023, J.D. Dig leg.; HS1698.

#### Description.

**Figure 1.**
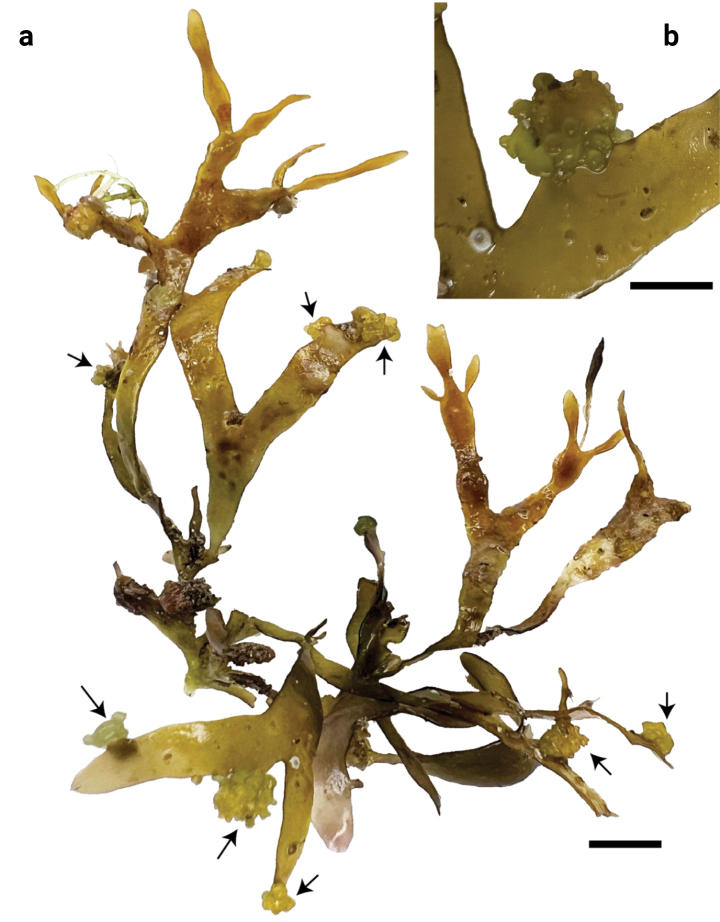
*Gracilariaphuquocensis*, THNHM2023-P-0090 (A585) **a** habit of a thallus **b** a branch portion of the host infected by an adelphoparasite resembling *Gracilariababae* (arrows), Scale bar: 5 mm.

Thallus (Fig. 1a) reddish-brown to yellowish-red, a foliose, erect solitary or clustered, dichotomously branched blade with entire margins, 1.5–5 cm tall, 1–3 mm wide and 0.2–0.3 mm thick. Thallus arising from a short cylindrical stipe that is attached to the substrate by a rhizoidal base. Thallus multiaxial, consisting of cortex and medulla. The cortex comprises up to two layers of pigmented globular cells and the medulla is composed of 2–4 unpigmented spherical or ovoid cells. Reproductive structures were not observed.

#### Observation of an adelphoparasite.

The adelphoparasites are parasites that are taxonomically closely related to their host species, which represent the majority of red algal parasites (Maren et al. 2017). Some specimens were infected by an adelphoparasite resembling *Gracilariababae* (Yamamoto) P.-K. Ng, P.-E. Lim et S.-M. Phang (Fig. 1b), although the identification of this parasite requires confirmation based on DNA. It formed amorphous swellings scattered along the margins of the blades. Individuals were up to 5 mm in diameter and almost the same colour (yellowish) as that of the host.

**Figure 2.**
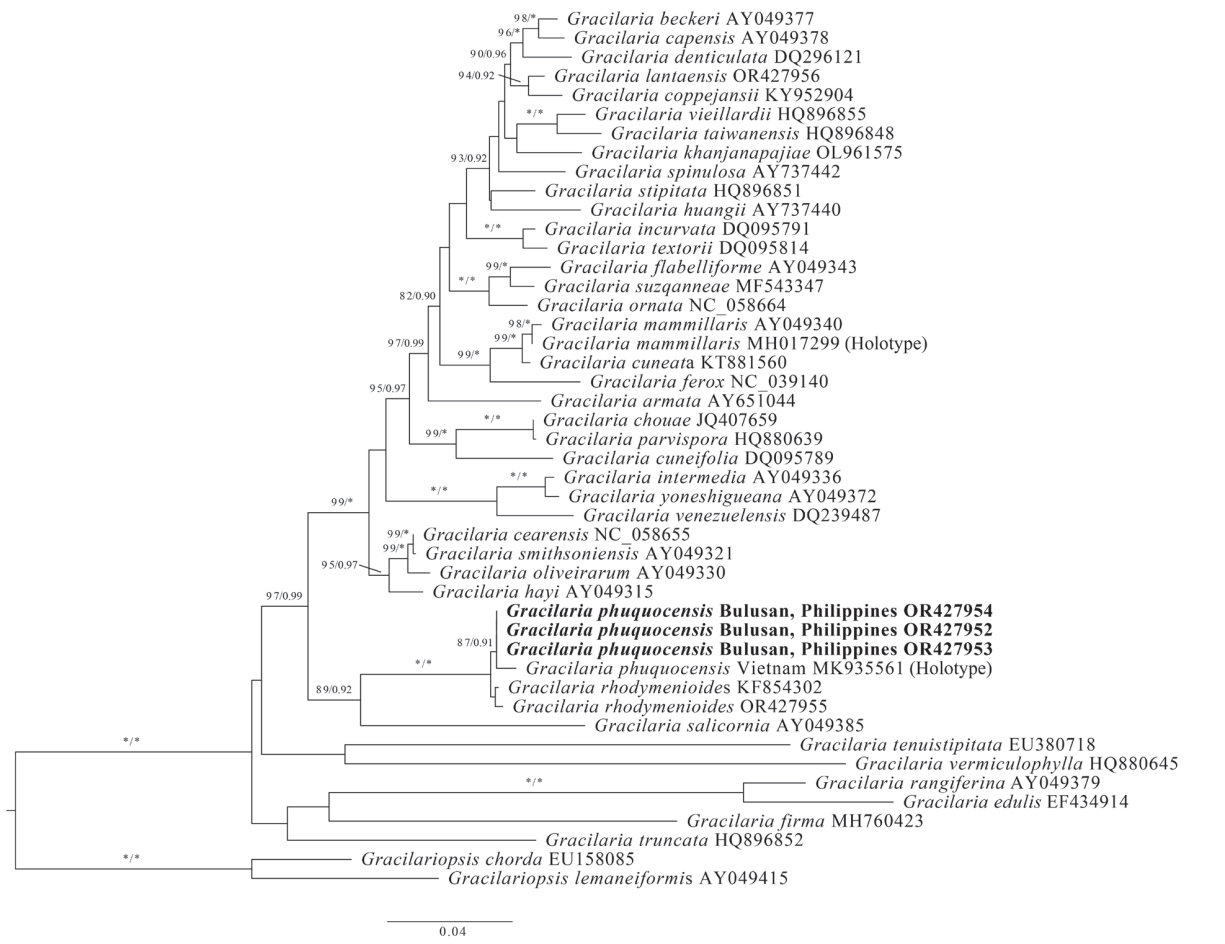
Maximum Likelihood (ML) tree, based on partial *rbc*L gene sequences showing the position of *Gracilariaphuquocensis* (bold letter) from Bulusan, Sorsogon, Philippines. ML bootstrap values (left) and Bayesian posterior probabilities (right) are indicated at the nodes. Bootstrap values of > 80% for ML and > 0.90 for BI are presented and full support are indicated by asterisk (*).

The *rbc*L sequences from the Philippine specimens of *G.phuquocensis* were closely related to that of the holotype (GenBank accession: MK935561) from Vietnam, as indicated by high support values (ML = 87%, BI = 0.91) in Fig. 2. The pairwise sequence differences amongst the Philippine specimens and the Vietnamese holotype ranged from 0.43% to 0.51%. The molecular data indicate that the Philippine *G.phuquocensis* is a distinct population, suggesting divergence due to geographical separation.

## ﻿Discussion

*Gracilariaphuquocensis* from Vietnam had been previously identified as *Gracilariamammillaris* (Montagne) M. Howe. However, *G.mammillaris* is considered to have a wide distribution in the western Atlantic (Gurgel et al. 2004), extending to occurrences in Venezuela and Brazil (Hardesty and Freshwater 2018), and a previous study by Le et al. (2020) as well as our recent research (Fig. 2) indeed confirmed that the Vietnamese material was phylogenetically distinct from *G.mammillaris*. The recognition of *G.phuquocensis* as a distinct taxon helps to provide a better base line for comparing Vietnamese specimens of *Gracilariacuneifolia* (Okamura) I.K. Lee & Kurogi, *Gracilariayamamotoi* Zhang & B.M. Xia and *Gracilariarhodymenioides* A.J.K. Millar, as these *Gracilaria* species are representative of taxonomically challenging complexes. While the current Philippine records only confirm the presence of *G.phuquocensis* amongst these flattened gracilarioids, some or all of the other species may be present in the area, but not recognised on morphological characters alone, especially since the macroalgal flora in the Philippines and Vietnam has high similarities in terms of species composition (Nguyen et al. 2013).

The presence of an adelphoparasite resembling *Gracilariababae* observed for the first time in *G.phuquocensis*, increases the number of gracilarioid species from which such neoplastic parasites (sensu Salomaki and Lane 2019) have been reported. *Gracilariababae* is only known to be associated with *Gracilariasalicornia* (C.Agardh) E.Y. Dawson (Yamamoto 1986) and an unidentified species of *Gracilaria* reported as *Hydropuntia* sp. (Ng et al. 2014). However, until molecular studies are undertaken, the identity of the species on *G.phuquocensis* remains unconfirmed.

Our study suggests that the Philippines seems to present hidden diversity of gracilarioid species, at least in the southeast Asia (Phang et al. 2021; Saengkaew et al. 2022; Nguyen et al. 2023). The latest reports for gracilarioid species in the Philippines prior to this study had six new records (Lastimoso and Santiañez 2021). Adding *G.phuquocensis*, this study brings the number of recorded Philippine gracilarioids to 46 taxa.

Yet another concerning issue is that the taxonomic accuracy underpinning gracilarioid names need updating (see Lyra et al. 2021). More attention must also be given to species that remain to be described or are overlooked. The number of gracilarioid species thus far reported from the Philippines (see Lastimoso and Santiañez 2021) appears to be inflated. Over the years, several workers, many of whom were working at a time when taxonomic concepts were based on doubtful morphological characters, have added records to the local flora, often using names for species found outside the Indo-Pacific Region. For example, *Gracilariavenezuelensis* W.R.Taylor was reported from the Philippine (Westernhagen 1973) and subsequently included in a number of Philippine catalogues and checklists without being verified. However, it was considered to be restricted to the Caribbean Basin (Gurgel et al. 2004), although there is always the possibility of the presence of non-indigenous species in the Philippine flora. This underscores the need to check available vouchers and to add records in the future that are backed by morphological and/or molecular data.

## Supplementary Material

XML Treatment for Gracilaria phuquocensis

## ﻿Additional information

### Author ORCIDs

Richard V. Dumilag https://orcid.org/0000-0002-7590-0009

Lawrence M. Liao https://orcid.org/0000-0001-5484-6560

Aki Kato https://orcid.org/0000-0001-6124-9676

Juliet Brodie https://orcid.org/0000-0001-7622-2564

Narongrit Muangmai https://orcid.org/0000-0001-7954-7348

### Data availability

All of the data that support the findings of this study are available in the main text.
